# Using the New CellCollector to Capture Circulating Tumor Cells from Blood in Different Groups of Pulmonary Disease: A Cohort Study

**DOI:** 10.1038/s41598-017-09284-0

**Published:** 2017-08-25

**Authors:** Yutong He, Jin Shi, Gaofeng Shi, Xiaoli Xu, Qingyi Liu, Congmin Liu, Zhaoyu Gao, Jiaoteng Bai, Baoen Shan

**Affiliations:** 1 Cancer Institute, The Fourth Hospital of Hebei Medical University/The Tumor Hospital of Hebei Province, Shijiazhuang, Hebei 050011 P.R. China; 2 Department of Radiology, The Fourth Hospital of Hebei Medical University/The Tumor Hospital of Hebei Province, Shijiazhuang, Hebei 050011 P.R. China; 3 Follow-up Centre, The Fourth Hospital of Hebei Medical University/The Tumor Hospital of Hebei Province, Shijiazhuang, Hebei 050011 P.R. China; 4 Department of Thoracic Surgery, The Fourth Hospital of Hebei Medical University/The Tumor Hospital of Hebei Province, Shijiazhuang, Hebei 050011 P.R. China; 5 Hebei Viroad Biotechnology Co., Ltd, Shijiazhuang, 050011 Hebei China

## Abstract

Circulating tumor cells (CTCs) are promising biomarkers for clinical application. Cancer screening with Low-Dose Computed Tomography (LDCT) and CTC detections in pulmonary nodule patients has never been reported. The aim of this study was to explore the effectiveness of the combined methods to screen lung cancer. Out of 8313 volunteers screened by LDCT, 32 ground-glass nodules (GGNs) patients and 19 healthy volunteers were randomly selected. Meanwhile, 15 lung cancer patients also enrolled. CellCollector, a new CTC capturing device, was applied for CTCs detection. In GGNs group, five CTC positive patients with six CTCs were identified, 15.6% were positive (range, 1–2). In lung cancer group, 73.3% of the analyzed CellCollector cells were positive (range, 1–7) and no “CTC-like” events were detected in healthy group. All CTCs detected from GGNs group were isolated from the CellCollector functional domain and determined by whole genomic amplification for next-generation sequencing(NGS) analysis. NGS data showed that three cancer-related genes contained mutations in five CTC positive patients, including KIT, SMARCB1 and TP53 genes. In four patients, 16 mutation genes existed. Therefore, LDCT combined with CTC analysis by an *in vivo* device in high-risk pulmonary nodule patients was a promising way to screen early stage lung cancer.

## Introduction

Lung cancer is an aggressive and heterogeneous disease, which has become the most common cancer for several decades and is the first leading cause of death from cancers worldwide^1, 2^. In most western countries, lung cancer incidence and mortality rates are decreasing in men and plateauing in women^3, 4^; however, rates of lung cancer are still increasing in China, and there were approximately 652,800 new cases and 597,200 deaths in 2015, accounting for 35.78% and 37.56% worldwide, respectively, which makes it a major public health problem^5^. Lung cancer remains lethal in both developing and developed countries, with a five-year survival rate generally lower than 20%^6^. A key point is that two in three lung cancer patients have an advanced stage at initial diagnosis, and the opportunity for potentially curative interventions is lost^7^. Therefore, minimal damage, effective and convenient detection at early stages of lung cancer is particularly necessary.

Screening with Low-Dose Computed Tomography (LDCT) is one tool that may increase the early detection and reduce the mortality in lung cancer^8^. LDCT is the most common used in high-risk candidate of lung cancer in the world wide^9^. Recent results from the National Lung Screening Trial (NLST) demonstrated that there was a 20% reduction in lung cancer-related deaths among patients who underwent an annual screening with LDCT compared to screening with chest radiographs^10^. Though LDCT is rapidly evolving, commonly available and uses advanced imaging technology, it still suffers from limitations, such as the high cost and inability to provide an accurate early diagnosis^11^. About 5–10% ground-glass nodules (GGNs) patients will be lung cancer in future^12, 13^. Recently, the U.S. Preventive Services Task Force recommendation statement (USPSTF) suggested annual LDCT-screening for lung cancer in high-risk individuals and stressed the need for more research of the use of biomarkers to complement LDCT screening^14, 15^. Biomarkers, which reflect the chemical and biological substances presented in the tumor, can assist in early diagnosis, classification and treatment of tumors^16^. In addition, the abnormity of the peripheral blood biomarkers in patients who have no clinical symptoms often emerge earlier than radiographic abnormalities^17–19^. Hence, it is necessary to explore new diagnostic and predictive biomarkers that could cover the shortages of the imaging techniques^20, 21^.

Circulating tumor cells (CTCs) are tumor cells that have disseminated from primary and metastatic sites, and circulate in the bloodstream. CTCs were considered as a biomarker of liquid biopsy approach for the early diagnosis of lung cancer^22^. The most difficult challenge facing the study of CTCs is the sensitive enrichment of these rare cells, which typically present as a single tumor cells against a background of millions of white blood cells^23^. Over the past decades, several *in vitro* methodological approaches to isolate and detect rare CTCs in the peripheral blood of patients have been reported, including flow cytofluorometry, image-based immunological approaches, fluidic microchip technology and PCR methods^24–26^. The widely used CTC isolation technique is based on the use of magnetic beads coated with antibodies against the epithelial cell adhesion molecule (EpCAM) to capture EpCAM-expressing cells, followed by immunostaining of the captured cells. The cell-enumeration results are always expressed as the number of CTCs per the maximum of 7.5 ml blood^27^. The limitation of all current *in vitro* techniques is the small blood volume available for CTCs enrichment, which is related to the relatively low sensitivity of this approach^28^.

To overcome the limitations of small blood sample volumes of the *ex vivo* CTCs isolation techniques, the CellCollector, which is the first *in vivo* CTC isolation product worldwide, was invented by GILUPI^29^. It is a structured and functionalized medical wire, which offers the opportunity of capturing CTCs from the circulating blood of patients under the largest blood flow volume. It was approved by Conformite Europeenne (CE) in 2012. Captured CTCs were identified based on the intensity of the cytokeratin immunofluorescence signal. Clinical application data have shown that the CellCollector has a high detection rate in several cancers, including lung cancer, prostate cancer, breast cancer and neuroendocrine tumors^29–33^. However, there has been no research about the CellCollector combined with LDCT screening lung cancer at an early stage until now. In this study, we applied the new CellCollector technique to detect CTCs in a high risk population to explore the potential application in early stage screening and diagnosis of lung cancer patients.

## Results

### Characteristics of the study individuals

A total of 8313 asymptomatic volunteers, including 4347 males and 3966 females, attended the lung cancer-screening program by LDCT detection. The median age of all volunteers was 52-years-old (range, 32–85). A total of 8005 volunteers were married, and 5551 volunteers had the education background of junior college and above. The number of the volunteers with occupations as workers, civil servants and other professions were 5294, 1964 and 1055, respectively (Table 1). Of the volunteers, 84.10% (6991/8313) were diagnosed with having “no significant abnormal findings observed in the thorax” and 1322 were diagnosed with “pulmonary nodules”. Among them, 88 were diagnosed as “ground-glass nodules (GGNs)”, accounting for 6.66% of all pulmonary nodules patients (Table 2).

**Table 1:** The basic information of the volunteers detected by LDCT.

	No			X^2^	P value
	Nodules	No Nodules	Total		
**Gender**				1.01	0.32
Male	708	3639	4347		
Female	614	3352	3966		
**Marriage Status**				3.64	0.06
Married	1261	6744	8005		
Unmarried	61	247	308		
**Education Status**				6.89	0.01
Below Senior high school	398	2364	2762		
Junior college and above	924	4627	5551		
**Occupation**				5.19	0.08
Worker	875	4419	5294		
Civil servant	300	1664	1964		
Else	147	908	1055		
**Smoking history**					
Never smoker	5247	935	6182	26.90	<0.001
Former smoker	1365	278	1643		
Current smoker	299	98	397		
**Drinking history**					
Yes	4883	844	5727	19.48	<0.001
No	1786	413	2199		
**Family history of cancers**					
Yes	4152	710	4862	8.07	0.005
No	2739	558	3297

**Table 2:** The diagnosis results of LDCT.

Classification	Male		Female		Total N(%)
	N	(%)	N	(%)	
Normal	3639	43.77	3352	40.32	6991 (84.10)
Micronodules (≤5 mm)	427	5.14	346	4.16	773 (9.30)
Small nodules (>5 mm, ≤10 mm)	181	2.18	163	1.96	344 (4.14)
Solid nodules (>10 mm)	53	0.64	33	0.40	86 (1.03)
GGNs	34	0.41	54	0.65	88 (1.06)
Mass lesions	13	0.16	18	0.22	31 (0.37)
total	4347	52.29	3966	47.71	8313 (100)

**Figure 1:**
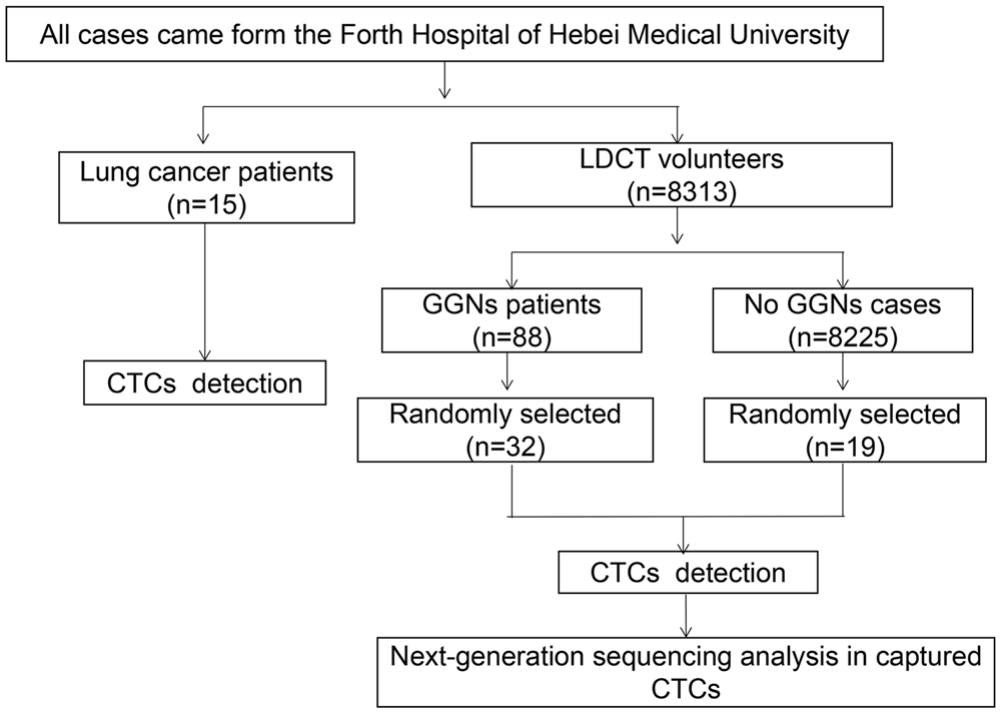
The Flowchart showing the technological process of CTCs detection.

32 patients who were randomly selected from the 88 GGNs patients, 15 lung cancer patients and 19 healthy volunteers had the CTCs detection performed by the CellCollector (Fig. 1 and Table 3). A total of 26.7%, 28.1% and 21.1% in three groups had history of smoking, respectively. There were 8, 13 and 9 individuals who had family histories of cancer in the three groups. All of the variables in the three groups had no significant differences.

**Table 3:** The characteristics of the study groups.

Classification	Lung cancer group	GGNs group	Negative control group
**Number**	15	32	19
**Gender**			
Male	2	14	4
Female	13	18	15
**Median age (range)**	54 (36–79)	52 (36–83)	48 (41–78)
**Cigarette history**	4	9	4
**Family history of cancers**	8	13	9
**Number persons of positive outcome**	11	5	0

### Overview of CellCollector and CTC identification

**Figure 2:**
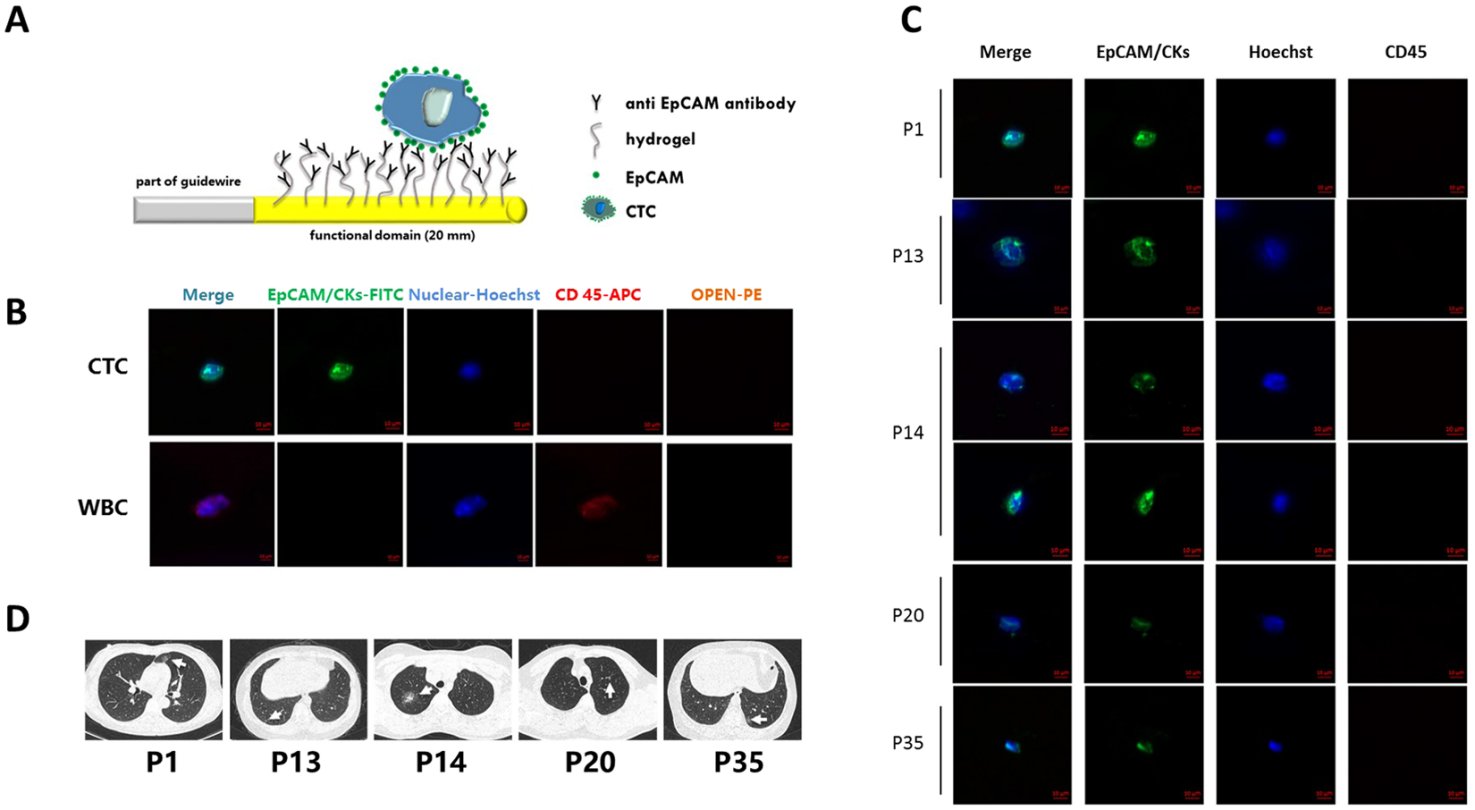
Circulating tumor cells capture and identification *in vivo*. (**A**) Schematic overview of the wire and the *in vivo* application. The wire is coated with anti-EpCAM antibodies and placed into the arm vein of cancer patients for 30 minutes. During the application EpCAM-positive cells bind to the device. (**B**) The tumor cells are stained for EpCAM and keratins. Nuclear counterstain is done using Hoechst33342. CD45 staining is necessary to classify false positive events (leukocytes). Scale bars, 10 μm. (**C**) Images of CTCs isolated with the CellCollector. Tumor cells were identified as EpCAM- and/or pan-keratin-positive (green) and CD45-negative (red) events. Hoechst33342 (blue) was used for nuclear counterstain. Scale bars, 10 μm. (**D**) LDCT scan images of five CTC-positive patients. GGNs, irregularly shaped solid nodule mixed GGNs and a solitary GGNs were shown with arrows indication.

The surface of the functional domain of the CellCollector was coated with anti-EpCAM antibodies and inserted into the arm vein of each subject for 30 minutes. EpCAM-positive cancer cells were captured by an antibody that exists on the functional domain of the CellCollector (Fig. 2A). The isolated tumor cells were stained for EpCAM and keratins. Nuclear counterstain was performed using Hoechst33342. CD45 staining was used to identify false positive events (white blood cells). EpCAM/CK-positive, nuclear positive and CD45-negative cells were identified as CTCs, and EpCAM/CK-negative, nuclear positive and CD45-positive cells were identified as leukocytes. Tumor cells were identified as EpCAM- and/or pan-keratin-positive (green) and CD45-negative (red). The representative images of CTC and WBC are shown in Fig. 2B.

**Figure 3:**
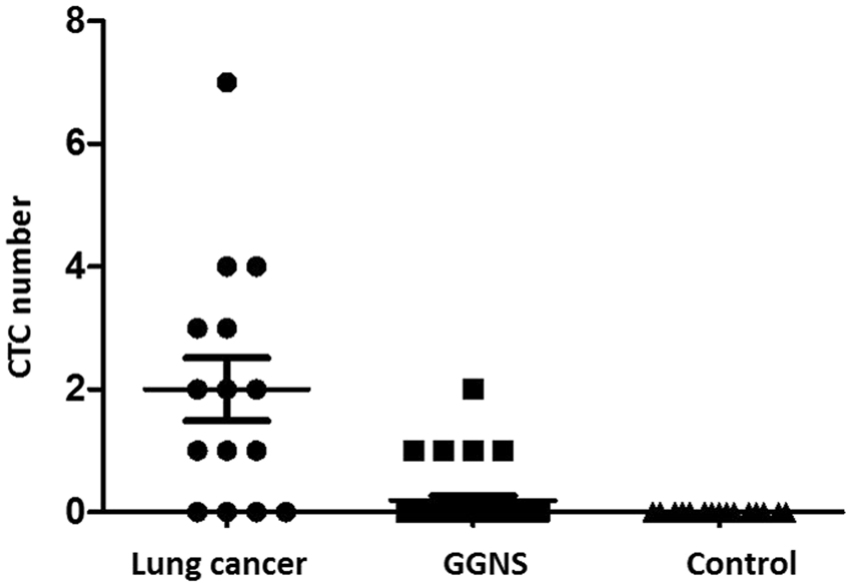
Detection rate of CellCollector in lung cancer patients, GGNs patients and benign patients. Median with interquartile range was shown.

In this study, based on CTC identification criterion, in the GGNs group, six EpCAM/CK-positive CTCs were identified from five patients. The LDCT scans of the five patients and the images of the six CTCs are shown in Fig. 2C,D. 15.6% (5/32) of the observed CellCollector patients were positive for ≥1 CTC (range, 1–2) in the GGNs group. The EpCAM/CK staining, nuclear staining and CD45, anti-leukocyte antibody staining results were exhibited. For the positive group, 73.3% (11/15) of the patients analyzed by the CellCollector were positive for ≥1 CTC (range, 1–7) in advanced lung cancer. No “CTC-like” events were detected in the healthy control group (n = 19; Fig. 3).

### NGS analysis of CTC on CellCollector

**Figure 4:**
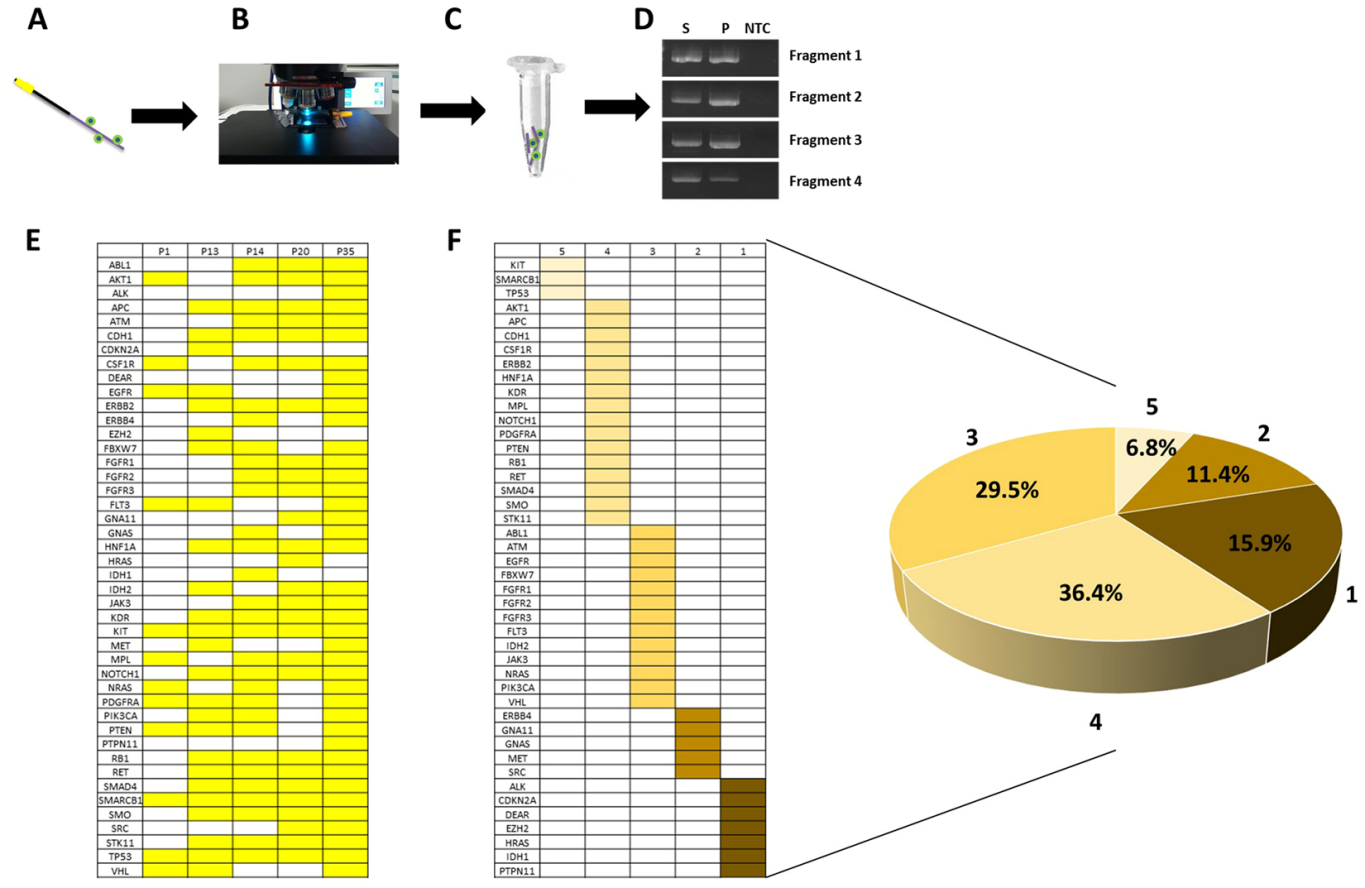
Next-generation sequencing analysis in captured CTC. (**A**–**D**) Cells isolated with the CellCollector were analyzed microscopically by means of immunofluorescence staining according to the staining procedure in the clinical setup (**A**,**B**). Cell Collector was fragmented and subjected to WGA (**C**). After WGA, quality of WGA DAN was analyzed with PCR assay. Four specific fragments were amplificated with WGA DNA, PBMC and ultrapure water. S, indicated WGA DNA; P indicated positive control; NTC indicated ultrapure water. (**E**) Gene mutations were analyzed with Hotspot panel v2 in five patients’ CTCs. Different colors indicated specific mutation sites in patients. (**F**) Cancer-related mutation genes were shown with form and pie chart. Different colors indicated different proportion. Numbers one to five indicated gene mutation exist in patient number.

To obtain genetic information of captured CTCs, we analyzed gene mutations of CTCs with high-throughput sequencing methods. Cells isolated with the CellCollector were observed microscopically by means of immunofluorescence staining according to the staining procedure. The functional domain of the CellCollector was fragmented and performed whole genomic amplification (WGA). The quality of WGA DNA was analyzed using PCR assay with four primer pairs (Fig. 4A–D).

Gene mutations were analyzed with Hotspot Panel v2 in five GGNs patients’ CTCs (Fig B). In total, 44 cancer-related genes existed in mutations in those analyzed CTCs. Mutations in the KIT, SMARCB1 and TP53 genes were found in five CTCs-positive patients. In four patients, 16 gene mutations existed. In three patients, 13 gene mutations existed. Approximately 27.3% (12/44) of genes existed in two or one patients (Fig. 3F).

## Discussion

Here, we provide a potential method for early lung cancer screening with LDCT and CTC detection. We first addressed the clinical needs of sensitivity screening techniques for identification of early lung cancer with LDCT analysis and how to detect CTCs with the CellCollector, a new *in vivo* method to isolate CTCs. Importantly, the captured CTCs can easily separate for downstream molecular analysis, such as digital PCR or NGS analysis, to provide more genetic information.

The high incidence and mortality rates for lung cancer coupled with a very low five-year survival rate makes late diagnosis a major obstacle. Survival of patients undergoing lung resection is greater than 80%, suggesting that early detection and diagnosis of cancers will greatly reduce the mortality^34, 35^. Recent studies suggest that LDCT could provide an alternative strategy to improve lung cancer diagnoses and outcomes^8^. However, a high false-positive rate leads to subsequent follow-up and invasive testing, which has its own associated psychological harms such as anxiety and depression^36–38^. Moreover, in terms of the GGNs patients, it is difficult to distinguish who the high-risk individual of lung cancer is. Because of these limitations, a non-invasive test with a high specificity for distinguishing the indolent disease from lung cancer patients is in high demand^39^.

In the recent decades, detection of serum tumor marker levels has become a way of improving the rate of early diagnosis of lung cancer^40^. Several serum tumor markers have been studied extensively, such as CEA, NES and LDH^41–43^. However, none have been demonstrated to provide clinical utility^39, 44, 45^. CTCs, known as a “liquid biopsy”, have recently emerged as a potential diagnostic biomarker in several cancers, including lung cancer, due to their fine sensitivity and specificity^46–48^. The CTC detection might make substantial contributions in the diagnostic workup of indeterminate lung lesions and avoids solid biopsy in selected cases, considering that up to 50% of resected indeterminate lung nodules are benign^49–52^. CTCs have already shown to be a potential clinical value in screening early stage lung cancer in COPD patients^53^. However, CTC detection rate is insufficient for clinical application, especially in early stage cancer screening, because of the disadvantages of *ex vivo* isolation methods.

So far, all the CTC detection methods cannot find CTC in 100%. Some patients have low-expression EpCAM CTC in their blood due to epithelial-mesenchymal transition (EMT). CellCollector captured CTC with EpCAM antibody on its functional surface. So, in this study, about 73% lung cancer patients were detected as CTC positive. Detection sensitivity has been increased with *in vivo* isolation methods, as the CellCollector overcomes blood sample limitation and possesses a high detection rate in several cancers^29, 54, 55^. Moreover, by coating a hydrogel layer on the functional domain surface, the CellCollector has a low background of unspecific white blood cells. Therefore, captured CTCs could be isolated using a special cutter for downstream molecular analysis with related CTC-specific DNA templates. Additionally, WGA of CTCs increased the genomic DNA amount for the NGS assay. With WGA methods, single CTCs could be analyzed with digital PCR or NGS methods^56^. This is the first time a study has determined the detection rate of pulmonary disease among the Chinese population. In this study, 15 lung cancer patients, 32 GGNs and 19 healthy controls were detected using CTCs via the CellCollector. Of the lung cancer patients, 73.3% had CTCs isolated. The detection rate of the CellCollector in lung cancer was similar with that of published references, and no CTC-positive patients were found in the healthy volunteer group, indicating the application of the CellCollector in this study is reliable. A detection rate of 15.6% was observed in the GGNs group. Recently, JAMA reported that after one year of follow-up, more than 90% of lung cancer cases diagnosed by LDCT were false-positives. LDCT combined with other biomarkers, such as the CellCollector, may be a more effective method for early stage lung cancer screening^12^.

Immunofluorescence staining with EpCAM/CK, CD45 and DAPI/Hoechst was the most widely used method for CTC identification. A large number of clinical studies have shown that EpCAM/CK-positive CTCs are clinically relevant to prognosis, therapeutic effect and diagnosis^31, 57, 58^. Here, six CTCs were analyzed with immunofluorescence staining from five GGNs patients, suggesting those CTC-positive patients have a high risk of lung cancer. Genetic information of CTC provided more clinical information compared with CTC counts^59^. In this study, NGS analysis showed that all of the CTCs had abnormal gene mutations, indicating that those cells possessed malignant tendencies. Over 70% of mutated genes existed in at least three patients, indicating some common mutation genes had a mutation trend. Based on the NGS data, we found that three gene mutations existed in those five patients, which included KIT, SMARCB1 and TP53. All of those three genes were important cancer related genes. KIT mutation could induce drug resistance and correlate with prognosis in lung cancer^60^. SMARCB1 was well studied in familial schwannomatosis, rhabdoid tumors and familial multiple meningiomas^61, 62^. However, few studies have been reported in lung cancer research. Here, we found that all of the CTC positive patients had SMARCB1 gene mutations in the CTC genome, suggesting that SMARCB1 may have a relationship in the early stage of lung cancer. TP53 was found not only as a prognosis marker in early stage lung cancer but also as an important event in cancer development^63^. Here, TP53 mutations, which frequently exist in early tumor cells, were found in the identified CTCs, showing that those CTCs may come from early stage tumor tissue. A large number of CTCs need to be sequenced to screen specific marker mutations, as screening markers may be a future clinical application.

Because of the low detection rate of CTC, screening early stage lung cancer with CTCs as a biomarker was rarely studied. In this study, we combined an LDCT and CTC assay to screen early stage lung cancer. In those 32 GGNs patients, five CTC-positive patients were detected. CTCs were identified with an immunofluorescence staining method, which was widely used in clinical diagnosis and studies. Moreover, the gene mutation profile was analyzed when the CTC identification was finished. Of the cancer-related genes, 44 were found to have mutations in the analyzed cells, indicating that EpCAM/CK-positive cells have a malignant tendency. Therefore, those CTC positive patients require careful follow-up observation. More follow-up data, including PET CT results, need to be collected in the future to confirm the combined screening results.

## Materials and Methods

### Study design and clinical information

During the three-year period, from January 2014 to December 2016, in the Fourth Hospital of Hebei Medical University, we conducted a single-center population-based screening program for lung cancer in the setting of annual medical examinations with LDCT. All GGNs patients and the healthy volunteers (negative control group) came from the 8313 volunteers. According to the diagnosis results of LDCT, all GGNs patients were screened out and 32 GGNs patients were randomly sampled to perform the CTC detection. The patients in the negative control group, who had no pulmonary nodules and no prior evidence of lung cancer, also accepted the same detection method as the other two groups. We also selected 15 patients who were diagnosed with lung cancer in the same period as the lung cancer group. All the lung cancer patients were confirmed by pathological examination. All of them were required to fill out the questionnaire, which included sex, age, smoking history and family history of cancer (Fig. 1).

### Ethics statement

All of the individuals in the study were approved by the Forth Hospital of Hebei Medical University (Shijiazhuang, Hebei, China) and were performed in accordance with the approved guidelines. Informed consent were obtained form all individuals in the study.

### LDCT and pathological classification

LDCT is offered to all individuals who joined the physical examination. All CT scans were performed on thin-slice (0.625 mm) scanners from different manufacturers with variable numbers of detectors (16e320), from lung apex to base without contrast enhancement. The CT scanners included the GE LightSpeed 16 Slice CT scanner and the GE BrightSpeed 16 Slice CT scanner. All scans were obtained using a low-dose regimen, with the machine set at 120 kVp, 9 (15 mA/0.6 s) or 21 (35 mA/0.6 s) mAs, 1.5:1 pitch ratio, and a 0.6-second rotation time. The effective radiation dose ranged from 0.3 to 0.8 mSv.

We evaluated the attenuation (classified into micronodules, small nodules, solid nodules, GGNs and mass lesions) and size of nodules (according to the longest perpendicular diameter: 5 and 10 mm). Except GGNs and large mass lesions, other nodules were considered to be the solid. The solid nodules are defined as nodule densities completely equal or higher than pulmonary vessels. GGNs are defined as the focal nodular area of increased hazy lung attenuation through which normal parenchymal structures such as airways, interlobar septa, and vessels are visible, in contrast to the typical solid nodules that obscure lung parenchyma. Mass lesions should be considered malignant unless proven otherwise.

### *In vivo* application of the CellCollector

The CellCollector is comprised of stainless steel wire widely used in medicine. Antibodies to the epithelial cell surface antigen, EpCAM, were attached to a polycarboxylate/hydrogel, which was coated on a gold-plated layer. The CellCollector captured targets cells expressing EpCAM antigen on the cell surface of different cancer cell types. This antibody is regularly used in humans^29^.

Before the application of the CellCollector, a 20-G peripheral venous catheter was placed into the median cubital vein of the patient. The CellCollector was inserted into the vein through the catheter until the functional domain of the device was exposed to the blood flow. An IN-Stopper allowed its secure fixation to the intravenous cannula. The correct length of insertion was indicated by a mark on the distal part of the wire, which was not inserted into the cannula. The wire remained in the cubital vein during the 30 min application period, which is estimated at 1.5–3 liters^32, 64^.

### CTC identification

After the CellCollector application was finished, the CellCollector was gently washed in washing buffer, followed by incubation in PBS containing 2% (w/v) bovine serum albumin (BSA) for 30 min at room temperature. CTCs captured by the CellCollector were identified by immunocytochemical staining for EpCAM or cytokeratins 8, 18, and 19. Cells attached to the wire were incubated with a FITC-conjugated mouse monoclonal antibody directed to EpCAM (Acris, clone HEA125-FITC) and an APC-conjugated rabbit antibody raised against CD45 (Exbio, clone MEM-28-Alexa647). Cells were counterstained with the nuclear dye Hoechst33342 (Sigma). Intensity of the immunocytochemical staining of CTC was evaluated using an Axio Imager.A1m microscope (Zeiss, Jena, Germany) equipped with an AxioCam digital camera system and the AxioVision 4.6 software (Zeiss). EpCAM/cytokeratin-positive cells should had additional features, including a large cell body (diameter 10–50 μm), an irregular cell shape, a large irregularly shaped nucleus, and a high nuclear to cytoplasmic ratio. Cells were enumerated on each CellCollector by an operator who was blinded to the clinical information of the patients.

### CTC separation and whole genomic amplification

After CTCs were identified with IF staining, the positive cells were relocated and cut with a special cutter under a microscope. The cells and CellCollector fragments were collected together into an EP tube to lyse cells and amplify genomic DNA. The whole genomic amplification was performed following the instructions of the commercial WGA kit (REPLI-g Single Cell Kit (Qiagen). Briefly, Buffer D2 was prepared. (The total volume of Buffer D2 given in the following table is sufficient for 12 reactions. If performing fewer reactions, store residual Buffer D2 at −20 °C. Buffer D2 should not be stored longer than 3 months). The cell sample should be brought up to 4 μl by Mg2 + -free, Ca2 + -free PBS. Then, 3 μl of Buffer D2 was added and mixed carefully by flicking the tube and centrifuging briefly. Incubate in a thermal cycler at 65 °C for 10 min. Add 3 μl of Buffer N, mix it carefully by flicking the tube and centrifuge briefly. Store on ice. Add 40 µl of master mix to each prepared 10-µl DNA sample. Mix carefully by flicking the tube and centrifuge briefly. Incubate at 30 °C for 6 h. 65 °C for 3 min to inactivate Discover-sc DNA Polymerase. Amplified gDNA was quantified with the Qubit and Nanodrop (Life technology). For panel NGS analysis, the Life proton system was used.

### Statistical analysis

Demographic variables were presented as the medians (range, minimum-maximum). Categorical variables were compared by Pearson’s chi-square test. A *P* value < 0.05 was considered statistically significant. All statistical manipulations were performed using the SPSS 21.0 for the Windows software system.

### Data Availability

The datasets generated during and analysed during the current study are available from the corresponding author on reasonable request.

## References

[1] Ferlay J (2015). Cancer incidence and mortality worldwide, sources, methods and major patterns in GLOBOCAN 2012. Int J Cancer..

[2] National Lung Screening Trial Research T (2011). Reduced lung-cancer mortality with low-dose computed tomographic screening. N Engl J Med..

[3] Islami F, Torre LA, Jemal A (2015). Global trends of lung cancer mortality and smoking prevalence. Transl Lung Cancer Res..

[4] Torre LA, Siegel RL, Ward EM, Jemal A (2014). International variation in lung cancer mortality rates and trends among women. Cancer Epidemiol Biomarkers Prev..

[5] Chen WQ (2016). Cancer statistics in China, 2015. CA Cancer J Clin..

[6] Allemani C (2015). Global surveillance of cancer survival 1995-2009, analysis of individual data for 25,676,887 patients from 279 population-based registries in 67 countries (CONCORD-2). Lancet..

[7] Chen CY (2016). Lung cancer screening with low-dose computed tomography, Experiences from a tertiary hospital in Taiwan. J. Formos Med Assoc..

[8] Zhao SJ, Wu N (2015). Early detection of lung cancer, Low-dose computed tomography screening in China. Thorac Cancer..

[9] Roberts H (2013). Screening high-risk populations for lung cancer: guideline recommendations. J Thorac Oncol..

[10] Eberth JM (2014). Lung cancer screening using low-dose CT, the current national landscape. Lung Cancer..

[11] Louis E (2016). Detection of Lung Cancer through Metabolic Changes Measured in Blood Plasma. J. Thorac Oncol..

[12] Redberg RF, O’Malley PG (2017). Important Questions About Lung Cancer Screening Programs When Incidental Findings Exceed Lung Cancer Nodules by 40 to 1. JAMA intern Med..

[13] Shin KE (2014). Subcentimeter lung nodules stable for 2 years at LDCT Long-term follow-up using volumetry. Respirology..

[14] He YT (2016). Detection of cancer specific mutations in early-stage non-small cell lung cancer using cell-free DNA by targeted sequencing. Int J Oncol..

[15] Birse CE (2015). Blood-based lung cancer biomarkers identified through proteomic discovery in cancer tissues, cell lines and conditioned medium. Clin Proteomics..

[16] Leidinger P (2010). Identification of lung cancer with high sensitivity and specificity by blood testing. Respir Res..

[17] Yu Z (2014). Prediction of lung cancer based on serum biomarkers by gene expression programming methods. Asian Pac J Cancer Prev..

[18] Fiorelli A (2015). Circulating Tumor Cells in Diagnosing Lung Cancer, Clinical and Morphologic Analysis. Ann Thorac Surg..

[19] Nakajima T, Yasufuku K (2013). Early lung cancer, methods for detection. Clin Chest Med..

[20] Earl J (2015). Circulating tumor cells (Ctc) and kras mutant circulating free Dna (cfdna) detection in peripheral blood as biomarkers in patients diagnosed with exocrine pancreatic cancer. BMC Cancer..

[21] Zander T (2011). Blood-based gene expression signatures in non-small cell lung cancer. Clin Cancer Res..

[22] Yu N, Zhou J, Cui F, Tang X (2015). Circulating tumor cells in lung cancer, detection methods and clinical applications. Lung..

[23] Paterlini-Brechot P, Benali NL (2007). Circulating tumor cells (CTC) detection, clinical impact and future directions. Cancer Lett..

[24] Ring AE, Zabaglo L, Ormerod MG, Smith IE, Dowsett M (2005). Detection of circulating epithelial cells in the blood of patients with breast cancer, comparison of three techniques. Br J Cancer..

[25] Nagrath S (2007). Isolation of rare circulating tumour cells in cancer patients by microchip technology. Nature..

[26] Simpson SJ (1995). Detection of tumor cells in the bone marrow, peripheral blood, and apheresis products of breast cancer patients using flow cytometry. Exp Hematol..

[27] Allard WJ (2004). Tumor cells circulate in the peripheral blood of all major carcinomas but not in healthy subjects or patients with nonmalignant diseases. Clin Cancer Res..

[28] Miyamoto DT, Sequist LV, Lee RJ (2014). Circulating tumour cells-monitoring treatment response in prostate cancer. Nat Rev Clin Oncol..

[29] Saucedo-Zeni N (2012). A novel method for the *in vivo* isolation of circulating tumor cells from peripheral blood of cancer patients using a functionalized and structured medical wire. Int J Oncol..

[30] Chudak C, Herrmann J, Lesser T (2016). 113P, Enumeration and molecular characterization of circulating tumor cells in lung cancer patients using the GILUPI CellCollector. J. Thorac Oncol..

[31] Gorges TM (2016). Enumeration and Molecular Characterization of Tumor Cells in Lung Cancer Patients Using a Novel *In Vivo* Device for Capturing Circulating Tumor Cells. Clin Cancer Res..

[32] Theil G (2016). The Use of a New CellCollector to Isolate Circulating Tumor Cells from the Blood of Patients with Different Stages of Prostate Cancer and Clinical Outcomes – A Proof-of-Concept Study. PloS one..

[33] Mandair D (2016). A comparison of CellCollector with CellSearch in patients with neuroendocrine tumours. Endocr Relat Cancer..

[34] Qiu R (2016). Planning and Implementation of Low-Dose Computed Tomography Lung Cancer Screening Programs in the United States. Clin J Oncol Nurs..

[35] Hoseok I (2015). & Je-Yoel, Cho. Lung Cancer Biomarkers. Adv Clin Chem..

[36] Stacey D (2011). Decision aids for people facing health treatment or screening decisions. Cochrane Database. Syst Rev..

[37] Harris RP (2014). The harms of screening, a proposed taxonomy and application to lung cancer screening. JAMA Intern Med..

[38] Volk RJ (2014). Feasibility of a patient decision aid about lung cancer screening with low-dose computed tomography. Prev Med..

[39] Ma S (2016). Multiplexed Serum Biomarkers for the Detection of Lung Cancer. . EBioMedicine.

[40] Zhang D, Ren WH, Gao Y, Wang NY, Wu WJ (2013). Clinical significance and prognostic value of pentraxin-3 as serologic biomarker for lung cancer. Asian Pac J Cancer Prev..

[41] Chu XY (2011). Diagnostic values of SCC, CEA, Cyfra21-1 and NSE for lung cancer in patients with suspicious pulmonary masses, a single center analysis. Cancer Bio Ther..

[42] Yang ZM, Ding XP, Pen L, Mei L, Liu T (2014). Analysis of CEA expression and EGFR mutation status in non-small cell lung cancers. Asian Pac J Cancer Prev..

[43] Ziaian B (2014). Association of high LDH and low glucose levels in pleural space with HER2 expression in non-small cell lung cancer. Asian Pac J Cancer Prev..

[44] Ilie M (2014). Current challenges for detection of circulating tumor cells and cell-free circulating nucleic acids, and their characterization in non-small cell lung carcinoma patients. What is the best blood substrate for personalized medicine?. Ann Transl Med..

[45] Esposito A, Criscitiello C, Trapani D, Curigliano G (2017). The Emerging Role of “Liquid Biopsies,” Circulating Tumor Cells, and Circulating Cell-Free Tumor DNA in Lung Cancer Diagnosis and Identification of Resistance Mutations. Curr Oncol Rep..

[46] Tanaka F (2009). Circulating tumor cell as a diagnostic marker in primary lung cancer. Clin Cancer Res..

[47] Ning N (2014). Improvement of specific detection of circulating tumor cells using combined CD45 staining and fluorescence *in situ* hybridization. Clin Chim Acta..

[48] Huang H (2016). Circulating tumor cells as a potential biomarker in diagnosis of lung cancer, a systematic review and meta-analysis. Clin Respir J..

[49] Karachaliou N, Mayo-de-Las-Casas C, Molina-Vila MA, Rosell R (2015). Real-time liquid biopsies become a reality in cancer treatment. Ann Transl Med..

[50] Han Y, Su C, Liu Z (2014). Methods for detection of circulating cells in non-small cell lung cancer. Front Biosci..

[51] Nurwidya F (2016). Circulating Tumor Cell and Cell-free Circulating Tumor DNA in Lung Cancer. Chonnam Med J..

[52] Krebs MG (2012). Analysis of circulating tumor cells in patients with non-small cell lung cancer using epithelial marker-dependent and -independent approaches. J Thorac Oncol..

[53] Ilie M (2014). “Sentinel” circulating tumor cells allow early diagnosis of lung cancer in patients with chronic obstructive pulmonary disease. PloS one..

[54] Alix-Panabieres C, Pantel K (2014). Challenges in circulating tumour cell research. Nat Rev Cancer..

[55] Kuske A (2016). Improved detection of circulating tumor cells in non-metastatic high-risk prostate cancer patients. Sci Rep..

[56] Do H (2016). Digital PCR of Genomic Rearrangements for Monitoring Circulating Tumour DNA. Adv Exp Med Biol..

[57] Braun S, Marth C (2004). Circulating tumor cells in metastatic breast cancer–toward individualized treatment?. N Engl J Med..

[58] Cristofanilli M (2006). Circulating tumor cells, disease progression, and survival in metastatic breast cancer. Semin Oncol..

[59] Krebs MG (2014). Molecular analysis of circulating tumour cells-biology and biomarkers. Nat Rev Clin Oncol..

[60] Lu HY (2012). Expression and mutation of the c-kit gene and correlation with prognosis of small cell lung cancer. Oncol Lett..

[61] Christiaans I (2011). Germline SMARCB1 mutation and somatic NF2 mutations in familial multiple meningiomas. J Med Genet..

[62] Hulsebos TJ (2007). Germline mutation of INI1/SMARCB1 in familial schwannomatosis. Am J Hum Genet..

[63] Lee SY (2015). The influence of TP53 mutations on the prognosis of patients with early stage non-small cell lung cancer may depend on the intratumor heterogeneity of the mutations. Mol Carcinog..

[64] Fortune JB, Feustel P (2003). Effect of patient position on size and location of the subclavian vein for percutaneous puncture. Arch Surg..

